# Effectiveness of mobile health‐based self‐management application for posttransplant cares: A systematic review

**DOI:** 10.1002/hsr2.434

**Published:** 2021-11-17

**Authors:** Sanaz Abasi, Azita Yazdani, Shamim Kiani, Zahra Mahmoudzadeh‐Sagheb

**Affiliations:** ^1^ Department of Health Information Management School of Health Management and Information Sciences, Shiraz University of Medical Sciences Shiraz Iran; ^2^ Clinical Education Research Center, Health Human Resources Research Center, School of Health Management and Information Sciences, Shiraz University of Medical Sciences Shiraz Iran; ^3^ Department of Health Information Management Health Human Resources Research Center, School of Health Management and Information Sciences, Shiraz University of Medical Sciences Shiraz Iran

**Keywords:** medication adherence, mobile health, posttransplant cares, self‐management

## Abstract

**Background and aims:**

Patients after transplantation need medical management for the rest of their lives, and self‐management seems to lead to greater adherence to medical standards, improve early physical changes, and increase patient empowerment. The main objective of this article is to systematic review of the consideration to mobile health applications (m‐Health apps) used in transplantation.

**Methods:**

A systematic search was conducted MEDLINE (through PubMed), Web of Science, Scopus, and Science Direct from inception to November 2020. The Preferred Reporting Items for Systematic Reviews and Meta‐Analysis (PRISMA) statement was used in this study. Comprehensive research was carried out using a combination of keywords and MeSH terms associated with m‐Health, empowerment, self‐management, and transplantation. Two independent reviewers screened titles and abstracts, assessed full‐text articles, and extracted data from articles that met inclusion criteria. Eligible studies were original research articles that included posttransplant care and mobile phone‐based applications to support self‐management and self‐care. Also, thesis, book chapters, letters to editors, short briefs, reports, technical reports, book reviews, systematic reviews, or meta‐analysis were excluded.

**Results:**

We divided all the reviewed articles into four categories, self‐management (medication adherence, adherence to medical regimen, and remote monitoring), evaluation, interaction, and interface; 37.5% of the studies were focused on lung transplantation. In 56.25% of the studies, medication adherence was considered because one of the main reasons for the rejection and graft loss is stated medication nonadherence. Also, 62.5% of the studies demonstrated that the use of m‐health improved medication adherence and self‐management in transplantation.

**Conclusions:**

The use of m‐Health apps interventions to self‐management after transplantation has shown promising feasibility and acceptability, and there is modest evidence to support the efficacy of these interventions. We found that m‐Health solutions can help the patient in self‐management in many ways after transplantation.

## 1 INTRODUCTION

Chronic illness is a condition that lasts for 1 year or more and requires constant medical attention.^1^ According to the World Health Organization (WHO), chronic diseases such as cardiovascular and respiratory diseases, cancer, and diabetes are the leading causes of death worldwide.^2^ On the other hand, transplantation, one of the most effective treatments for end‐stage organ failure,^3^ should also be considered a chronic disease because it requires lifelong follow‐up to manage concomitant diseases and prevent transplant loss.^4^ In 2018, a total of approximately 146 840 organ transplants were performed worldwide.^5^ Patients after transplantation need medical management for the rest of their lives, and they should engage in self‐care behaviors such as medication management, follow‐up medical appointments, and monitoring symptoms regularly. Although biological factors are essential for transplant survival, other factors, such as adherence to medication and medical care, are also influential.^6^ For example, one of the main reasons for the rejection and graft loss is stated medication nonadherence.^7^

Patients need daily care for daily life, communication with the treatment staff, scheduling an appointment with a doctor and going to the clinic, and adhering to medication. Furthermore, some of the conditions that may cause transplant rejection include infection, neoplasms, recurrence of significant diseases, nephropathy, metabolic syndrome, and surgical complications. Patients may also experience social psychological consequences such as social isolation, job disruption, financial crisis, and emotional burden.^8^ Most of the time, transplant recipients encounter many problems that they have to deal with on their own. It is necessary to change the routine of medical practices towards patients centered mode in this particular patient population. Therefore, self‐management is one way to manage or prevent potential health conditions after transplantation and has become increasingly critical for long‐term transplant survivors, which is believed to play a vital role in the improvement of quality of life and health status.^9^ Self‐management is the behaviors and activities that a person uses to practically manage the disease and manage the patient's physical or functional effects.^10^ Self‐management seems to lead to greater adherence to medical standards and improve early physical changes and increase patients' ability.^11^

Advances in information and communication technologies (ICTs) in health care have led to the development of m‐health, and it has revolutionized the provision of health services.^12^, ^13^ m‐Health to support patients with chronic diseases in self‐management has been widely considered in the last decade.^14^, ^15^ WHO defines m‐Health as a medical and public health approach supported by mobile devices, patient monitoring devices, personal digital assistants (PDAs), and other wireless devices.^2^ Statista website shows that the m‐Health market is growing and projected to exceed US$ 300 billion by 2025. Based on this information, it can be concluded that m‐Health has great potential in medical and public health approaches.^16^ Mobile phones have great potential to influence the management of chronic conditions worldwide due to their popularity, availability, portability, and technology capacity. m‐Health applications facilitate communication between patients and treatment staff, reduce chronic diseases costs, adhere to medical regimens, and influence patient outcomes.^14^, ^17^, ^18^

Due to the increasing popularity of m‐Health, some studies have reported the effect of m‐Health interventions on chronic diseases; for example, Fan and Zhao examined the effect of m‐Health on the outcomes of patients with chronic diseases and its limitations. This study showed a positive trend in m‐Health interventions for the management of chronic diseases.^19^ Badawy et al examined the evidence for the effectiveness of text messages and m‐Health applications to promote medication adherence in adolescents with chronic health conditions and stated that this approach is effective for promoting drug adherence in adolescents with chronic disease.^20^ Badawy et al also found that texting and mobile phone app interventions can correct adherence to preventive behavior in adolescents.^21^ Ramsey et al revealed the effectiveness of intervention m‐Health in improving the mental and physical health outcomes of young people undergoing cancer treatment and survivors of youth undergoing cancer treatment and child, adolescent, and young adult survivors of childhood cancer.^22^ Furthermore, Badawy et al measured e‐Health interventions for different outcomes of sickle‐cell disease self‐management, and their studies demonstrate the effect of e‐Health on the various components of sickle‐cell disease self‐management.^23^, ^24^

Although m‐Health interventions can help with patients' self‐management and to improve their care, few systematic studies have focused on the impact of using m‐Health technology on posttransplant self‐management. Therefore, this study was conducted to describe the main features of m‐Health interventions and their effectiveness on posttransplant self‐management outcomes to the following goals:

- To investigate the published studies on applying m‐Health in posttransplant care.
- To assess the efficacy of m‐Health in self‐management.
- To review the application quality evaluation scale.

## 2 METHODS

The current systematic review was based on the PRISMA checklist to ensure the inclusion of relevant studies.^25^

### 2.1 Literature search

We searched MEDLINE (through PubMed), Web of Science, Scopus, and Science Direct from inception to November 2020. Comprehensive research was carried out using a combination of keywords and MeSH terms associated with m‐Health, empowerment, self‐management, and transplantation. Table 1 shows a combination of keywords and MeSH terms used in our search.

**TABLE 1:** Keywords and search strategy

Keywords	Transplantation, mobile health, m‐Health, telemedicine, self‐management, self‐care
Search strategy	Transplantation AND (“mobile health” OR “m‐Health” OR “telemedicine”) AND (“self‐management” OR “self care”)
	“Organ transplantation” AND (“mobile health” OR “m‐Health “)

Articles were included if met the following criteria: (a) studies conducted on posttransplant care, (b) m‐Health applications interventions, and (c) only articles in the English language. Articles were excluded if they met the following criteria: (a) Self‐management applications without the use of m‐Health, (b) articles related to hematopoietic stem cell transplantation, bone marrow transplant, before solid organ transplant, etc., and (c) thesis, book chapters, letters to editors, short briefs, reports, technical reports, book reviews, review, or meta‐analyses.

### 2.2 Data extraction

**FIGURE 1:**
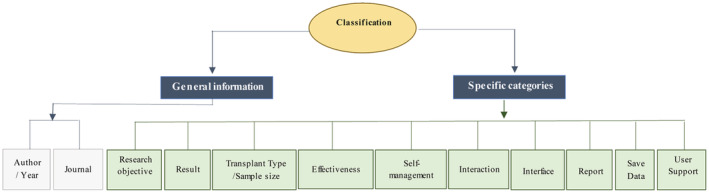
The classification of reviewed articles

After article retrieval, all titles and abstracts of articles were examined based on the main objectives, and reviewers selected relevant studies. Abasi and Kiani screened all titles and abstracts to find relevant articles. Articles that met our inclusion criteria were selected for full‐text review. Subsequently, full texts of relevant studies were screened thoroughly by two reviewers (Abasi and Kiani). Any conflicts were resolved by discussion with the senior authors (Yazdani and Mahmoudzadeh‐Sagheb). Lastly, after selected final articles, specific categories were considered to classify and analyze relevant articles. The categories were considered for the qualitative analysis of the articles present in Figure 1. Critical articles were summarized and entered into customized extraction forms based on these categories to diminish bias. Two authors (Abasi and Kiani) independently extracted the study characteristics from each article based on the classification. The information extracted by the researchers was reexamined to reach an agreement. The next reviewer (Yazdani and Mahmoudzadeh‐Sagheb) assessed and verified the extracted information. EndNote software was used for resource management. All synthesis and analysis were performed using SPSS v25.

## 3 RESULT

**FIGURE 2:**
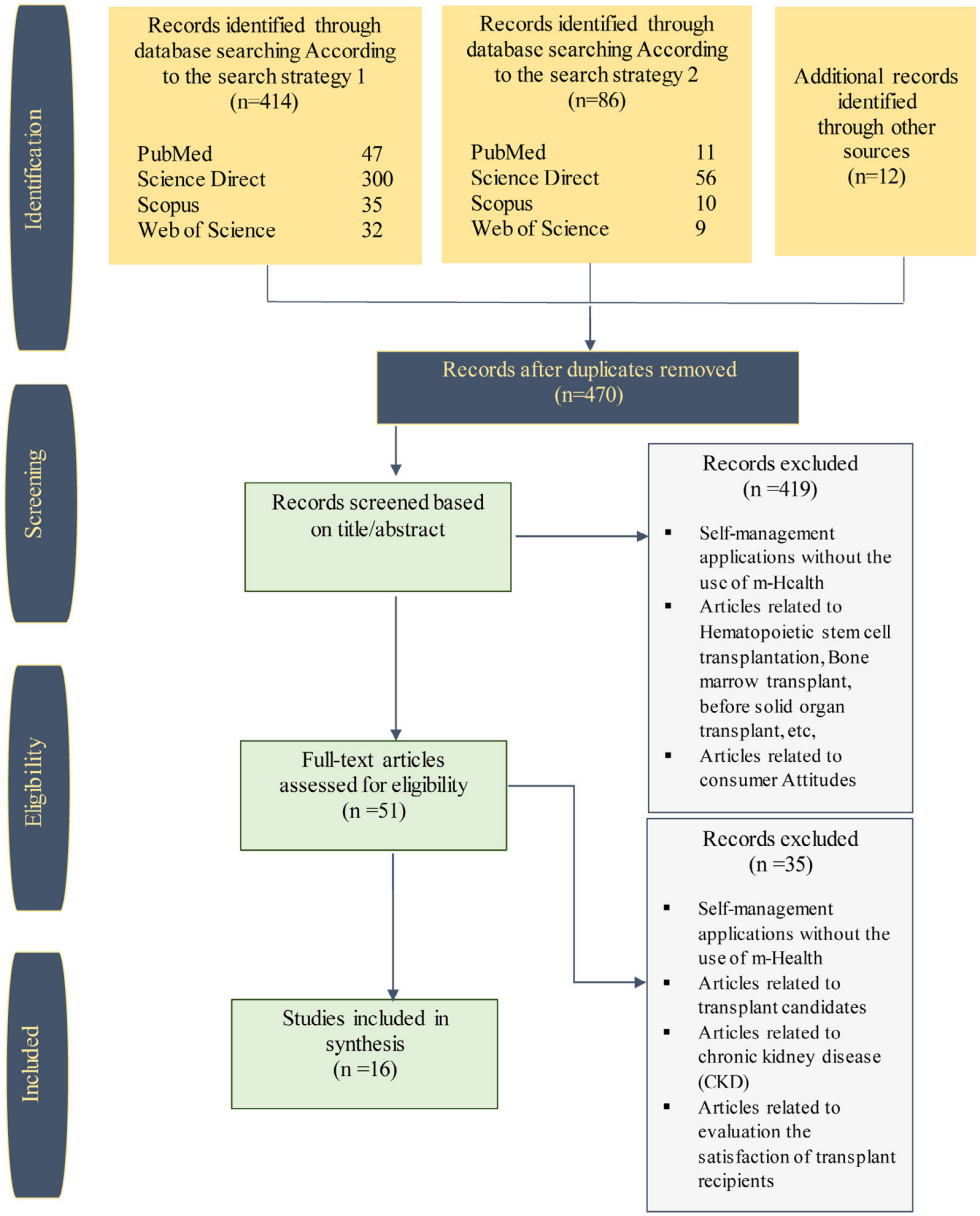
The flow diagram for the identification, screening, and eligibility of studies based on PRISMA

Based on our search strategy, 500 articles were retrieved. In addition, 12 studies were obtained by a simple search in Google Scholar. The procedure of screening articles based on the PRISMA method is displayed in Figure 2. After eliminating duplicate studies, 470 studies were retrieved. After evaluating the title and abstract of the studies, based on our inclusion criteria, 51 articles were selected to evaluate the full text. A total of 16 journal articles met our inclusion criteria. In this study, five categories, description of studies, self‐management, evaluation, interaction, and interface, were evaluated.

### 3.1 Description of studies

**FIGURE 3:**
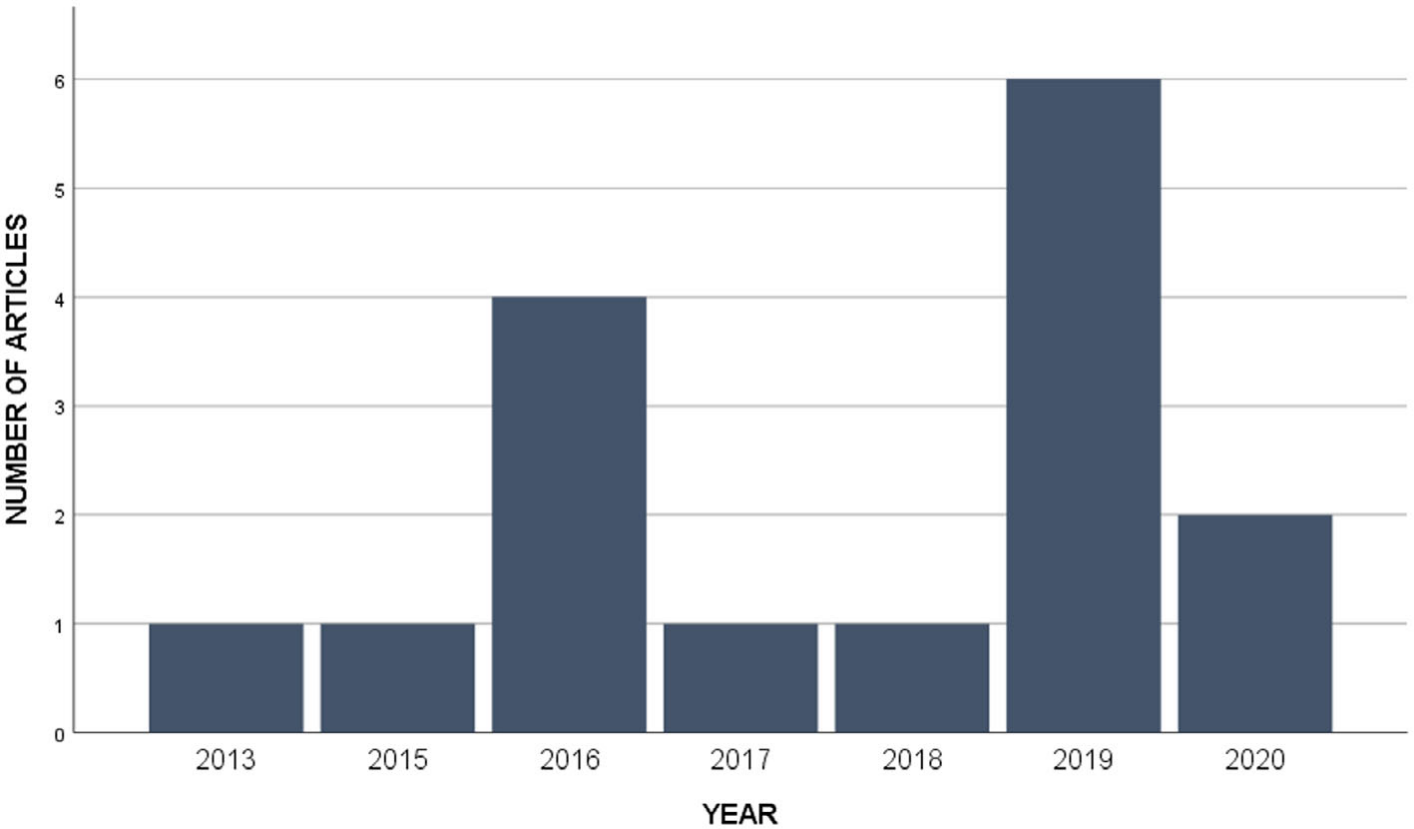
The distribution of papers by publication year

**FIGURE 4:**
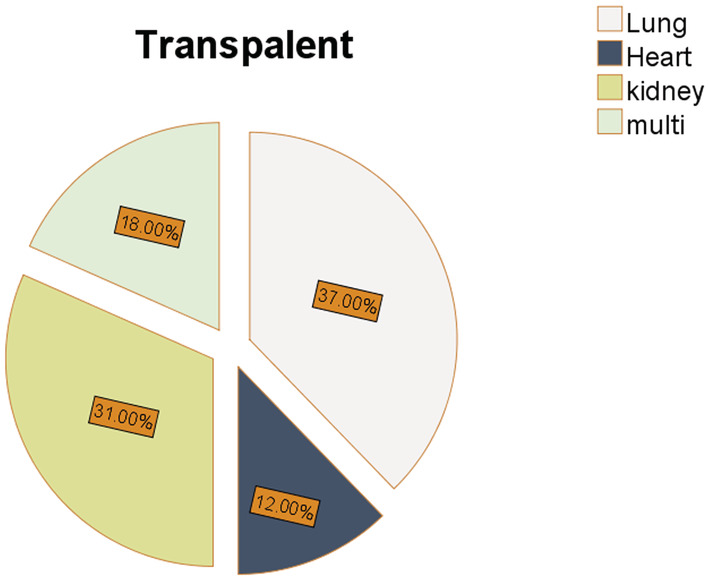
Frequency of studies according to organ transplantation

The distribution of studies by publication year is represented in Figure 3. Reviewed articles were diverse regarding the study design of the research. 50% of studies were randomized controlled trials (RCT), and most of the studies examined more than one outcome; 93.75% of studies reported the sample size. The sample size ranged from 7 to 201 with an average sample size of 80.6000 (SD = ±59.54806); 93.75% of studies reported the participant's age; in 37.5% of studies, participants were over 18 years old; in 18.75% of studies, a mean age of the participants was 57 years, and in 6.25% of studies, and participants' age span was 11 to 18 years. As shown in Figures 4, 68.75% of studies focused on kidney and lung transplant recipients, 12.5% of studies involved heart transplant recipients, and 18.75% of studies involving recipients of multiple transplants.

### 3.2 Self‐management

The posttransplant care starts immediately after transplantation surgery. Continuous follow‐up care should be provided for transplant patients to improve survival and outcomes. This critical care must continue after the patient is discharged from the hospital. All reviewed articles were categorized into three groups based on their objectives. These three topics include medication adherence, adherence to the medical regimen, and remote monitoring. In the following, the reviewed articles are described based on these three categories.

#### 3.2.1 Medication adherence

Medication adherence means nonobservance and deviation of the drug regimen and adverse effects on the prescribed regimen.^26^ Adherence to the medication regimen is especially important for immunosuppressive drugs in posttransplant patients. Due to the proven side effects of missing medication doses, there have long been intense efforts to increase medication adherence in transplant management. Among the included articles, 56.25% of studies were published on medication adherence.^27^

McGillicuddy et al^28^, ^29^, ^30^ performed studies to improve medication adherence (using automated reminders from an electronic medication tray) and blood pressure control in patients after kidney transplantation with uncontrolled hypertension.

Teen Pocket PATH is an m‐Health technology designed by Shellmer et al to promote adolescent independence in medication self‐management.^7^ Teen Pocket PATH is an m‐Health technology designed to promote adolescent independence in medication self‐management. Their goals were to support adolescents' independence, improve drug management, improve communication between adolescents and their primary caregiver, and remind as soon as possible to avoid missed and late doses. In Levine et al^27^ study, mobile application “Transplant Hero” has an interactive alarm and medication reminder for patients, it has also been used to provide educational content. The application uses a “Pebble Smart Technology” smart watch to display reminder notifications. Taber et al^31^ in their study mentioned the development, testing, and preliminary validation of an intervention using technology and under the supervision of a pharmacist. The goal was to improve the results and safety of the drug in kidney transplant recipients. The main reasons for poor outcomes after the kidney transplantation of medication safety issues include medication errors, medication nonadherence, and adverse drug events (ADEs). Gomis‐Pastor et al^32^ described the implementation of the “mHeart” model, and Gomis et al^33^ measured the therapeutical adherence improvement by mHeart. The mHeart platform is a mobile application and website used to facilitate patient communication and to record the timing of medication intake, drug interactions, vital signs, side effects, and symptoms. Cole et al study^34^ is a prospective research study for adult kidney transplant recipients, which aimed at improving the results of cardiovascular disease (CVD) risk factors and patient self‐efficacy and improving drug safety. They also described issues of racial segregation in transplant and the details of the intervention.

#### 3.2.2 Adherence to medical regimen

Three studies used personal assistant Pocket PATH for tracking health. Pocket PATH is one of the few m‐health programs for self‐management, which has been carefully developed, formally tested, and evaluated. Currently, its features are tailored to the posttransplant regimen for lung transplant recipients. These include alerts and reminders about taking immunosuppressive medications.^35^, ^36^, ^37^

Geramita et al^35^ used a Pocket PATH for lung transplant recipients in a randomized controlled trial (RCT). They measured the extent to which lung transplant recipients adhere to a medical regimen compared to lung transplant recipients receiving usual care during the first year after transplantation and other factors the potential risk for long‐term non‐compliance.

Rosenberger et al^36^ in a randomized controlled trial examined the association between those who had used Pocket PATH for 1 year and long‐term clinical outcomes, mortality, and bronchiolitis obliterans syndrome among 182 recipients.

In a randomized trial by DeVito Dabbs et al,^37^ 201 transplant recipients participated to compare effectiveness for promoting self‐management behaviors. They placed 99 people in the m‐Health intervention group and 102 in the usual care group. They also examined self‐care agency, re‐hospitalization, and mortality as secondary outcomes.

#### 3.2.3 Remote monitoring

Schenkel et al^38^ used Bluetooth‐based tablet technology in their study and, using this technology, transmitted patient vital signs information and respiratory parameters to real‐time transplant coordinators to improve transplant results. The platform also enables the communication between patients and providers and includes a comprehensive tutorial library with custom video content and daily reports. Lerret et al^39^ conducted a study based on the theory of individual and family self‐management and focused on the family self‐management of children transplant recipients at home. This pilot study performed a family self‐management intervention (myFAMI) using m‐Health technology to facilitate and support child family management and communication between family members and the healthcare team.

Jiang et al^40^, ^41^ intervened using Pocket PATH, an example m‐Health. The objectives of their study are the following: (a) To describe the use of mobile technology for self‐monitoring and acceptance rate of lung transplant recipients, and (b) To check the lung transplant recipients' follow‐up of decision support messages to report the recorded vital values and explore appropriate decision‐making predictors after technology support by reporting critical values during the first year after transplantation.

### 3.3 Evaluation

An important part of software development is quality evaluation. For example, user satisfaction is one of the evaluations that can be measured by examining the usability of the system^42^; 37/5% of the studies did not have an application quality evaluation scale,^27^, ^31^, ^33^, ^34^, ^37^, ^38^ and 31/25% of the articles evaluated acceptability and usability.^7^, ^28^, ^29^, ^30^, ^40^ Three studies used the PSSUQ or ASQ questionnaire for evaluation,^7^, ^40^, ^41^ and the other studies used surveys and interviews.^32^, ^39^

### 3.4 Interaction

According to Table 2, most research studies had interaction features, and we considered the interactive nature of m‐Health application feedback, alert, and reminder.

**TABLE 2:** The characteristics of reviewed articles

	Author/year	Journal/conference name	Research objective	Result	Transplant type /sample size	Effectiveness	Self‐management	Interaction (feedback/reminder/alarm)	Interface	Report	Save data	User support
1	Schenkel^38^/2020	*American Journal of Transplantation*	Improve outcomes in lung transplantation.	It is helpful in preventing readmission, reducing subsequent hospitalization days, and controlling hospital costs.	Lung transplantation/28	+	Remote monitoring	Inspirational messages/there are two types of alerts: email notifications for noncritical values but out of range, and phone alerts for critical values.	−	−	+	Face‐to‐face communication between patients and providers.
2	Gomis‐Pastor^32^/2020	*JMIR Cardio*	The main objective of this study was to describe the implementation of the mHeart model and to outline the main facilitators identified when conceiving an m‐Health approach.	Improve therapy management, patient empowerment, and patient‐provider interactions.	Heart transplantation/135	+	Medication adherence	Alerts, reminders, notifications, messages, and video calls.	−	+	+	The software was interactive with additional human support; a website was also designed for providers.
3	Geramita^35^/2020	*Transplantation*	To evaluate whether Pocket PATH had sustained effects on lung transplant recipients' medical regimen.	Showed better adherence to the medical regimen than lung transplant recipients receiving usual care during the first year post‐transplant.	Lung transplant/105 lung transplant recipients	+	Adhered to medical regimen	Its features include alerts and reminders, and decision support tools guiding patients on when to seek transplant team assistance.	+	−	+	Telephone
4	Lerret^43^/2019	*Journal of Pediatric Nursing*	myFAMI intervention with family members of pediatric transplant recipients and testing the initial effect on post‐discharge.	Facilitate patient/family‐nurse communication and family self‐management behaviors.	Heart, kidney, or liver transplant/include 40 family	+	Remote monitoring	The in‐app notification or prompt serves as a reminder.	−	+	−	Videoconference or a telephone
5	Levine^27^/2019	*American Journal of Surgery*	A mobile app (Transplant Hero) for medication adherence for transplant recipients.	Did not show an increase in medication adherence through the use of mobile health apps.	Adult kidney, pancreas, and/or liver transplant recipient/A total of 108 patients	−	Medication adherence	Interactive alerts and reminders of patients in the use of their medications as well as the preparation of educational content.	−	−	−	Not mentioned
6	McGillicuddy^28^/2019	*JMIR Research Protocols*	Improve medication adherence and sustain blood pressure control among kidney transplantation recipients.	Effectiveness of SMASK to improve medication adherence and blood pressure control in a group of hypertensive kidney transplant recipients.	Kidney transplantation/80	+	Medication adherence	Automated reminders from an electronic medication tray; proportional text messages and motivational feedback, guided by the self‐determination theory, and auto‐summary report for providers.	+	+	+	Email
7	Taber^31^/2019	*American Journal of Health‐System Pharmacy*	The development, testing, and preliminary validation of a technology‐enabled, pharmacist‐led intervention aimed at improving medication safety and outcomes in kidney transplant recipients.	The study demonstrated improved monitoring, management, and goal attainment for hypertension and diabetes control along with patient acceptability and the feasibility of the m‐Health system.	Kidney transplant/60	+	Medication adherence	Follow‐up survey/provider‐based feedback/reminders.	−	−	+	Text/telephone/email
8	Gomis^33^/2019	The *Journal of Heart and Lung Transplantation*	Measure the therapeutical adherence (TA) improvement by means of a personalized care program following heart transplant (HTx).	The effectiveness of the pharmaceutical interventions implemented through the mHeart tool was high.	Heart transplant32/	+	Medication adherence	Not mentioned.	−	−	+	Not mentioned
9	Cole^34^/2018	*Contemporary Clinical Trials Communications*	Demonstrate improved medication safety and CVD risk factor control in adult kidney transplant recipients at least one‐year posttransplant with a functioning graft.	The study will provide important and novel information regarding potential interventional methods to improve CVD risk factor control using innovative technology and pharmacist‐led interventions.	Kidney transplant/	+	Medication adherence	Not mentioned.	−	+	+	Mentioned
10	Rosenberger^36^/2017	*American Journal of Transplantation*	Examined Pocket PATH during the first year posttransplant.	Pocket PATH exposure had no direct effect on outcomes.	Lung transplant/182	+	Adhered to medical regimen	Alerts and reminders.	+	−	+	Mentioned
11	DeVito Dabbs^37^/2016	*American Journal of Transplantation*	The study was a randomized controlled trial comparing Pocket PATH and routine care. Outcomes were evaluated over 12 months.	Support for the potential benefits of Pocket PATH, an m‐Health intervention to promote self‐management.	Lung transplantation/201 recipients	+	Adhered to medical regimen	Automatically sends a reminder to the patient feedback.	+	+	+	Not mentioned
12	Shellmer^7^/2016	*Pediatric Transplantation*	The TPP prototype, an m‐Health application to promote medication adherence and strengthen communication on medication management between adolescents and primary caregivers, was developed and tested.	TPP generally easy to use and effective in prompting adolescents to adhere to their medications.	Adolescent solid organ recipients/7	++	Medication adherence	Warning system/reminder.	+	+	−	NOT mentioned
13	Jiang^41^/2016	*International Journal of Medical Informatics*	To examine the degree to which LTR followed decision support messages to report recorded critical values, and to explore predictors of appropriately following technology decision support by reporting critical values.	The majority of LTR responded appropriately to mobile technology‐based decision support for reporting recorded critical values.	Lung transplantation/96	+	Remote monitoring	Automatic feedback messages.	+	−	+	Messages
14	Jiang^40^/2016	*Applied Clinical Informatics*	To describe lung transplant recipients acceptance and use of mobile technology for health self‐monitoring during the first year posttransplantation.	Correlates were different for short‐ and long‐term use of mobile technology for health self‐monitoring in the first year posttransplantation.	Lung transplantation/96	++	Remote monitoring	Automatic feedback messages/reminding LTR to take action, including reporting the critical values to transplant clinicians.	+	−	+	A user support manual and a toll‐free number were given to LTR to call for help with technical problems.
15	McGillicuddy^29^/2015	*Progress in Transplantation*	Evaluate a mobile health pilot program to improve blood pressure and medication adherence.	Improvements seen in medication adherence and blood pressure control were promising.	Kidney transplant/19	++	Medication adherence	Electronically delivered medication and blood pressure reminder alerts, motivational and reinforcement messages for adherence, and the auditory and visual feedback of blood pressure control and medication adherence.	−	−	−	Not mentioned
16	McGillicuddy^30^/2013	*JMIR Research Protocols*	Assess the feasibility, acceptability, and preliminary outcomes of a prototype mobile health (m‐Health) medication and blood pressure (BP) self‐management system for kidney transplant patients with uncontrolled hypertension.	m‐Health intervention group exhibited significant improvements in medication adherence and significant reductions in clinic‐measured systolic blood pressures across the monthly evaluations.	Renal transplant/20	++	Medication adherence	Feedback/reminder	−	−	+	Text, email, or phone

### 3.5 Interface

In most studies, design criteria (use appropriate font sizes, use meaningful colors, use graphs, the contrast between text, and background, etc.) were not considered, but a few studies followed the principles of user‐centered design, and some used design criteria.^7^, ^28^, ^35^, ^36^, ^37^, ^40^, ^41^

## 4 DISCUSSION

In this research, we performed a study to investigate 16 related articles through a standard strategy. An initial objective of this study was to identify the published literature concerning solid organ transplantation and m‐Health.

Most studies have been performed on lung transplants. Lung transplant recipients are more likely to receive transplant complications than recipients of other solid organs, leading to increased mortality and increased use of health resources, so self‐management in this group is important.^37^ Pocket PATH is a well‐used and well‐known application in this field. Most studies demonstrated improved medication adherence^7^, ^28^, ^29^, ^30^, ^33^ and increased self‐care following the use of this technology, and patient empowerment, improved adherence to medical regimen, remote monitoring, and preventing readmission are examples of benefits obtained.^31^, ^32^, ^35^, ^38^, ^41^ Two studies mentioned that although m‐Health applications are a promising strategy, exposure had no direct effect on outcomes, and further research is required to determine how to best use this technology.^27^, ^36^

Medication management following solid organ transplants can be complex and burdensome. Patients often have multiple dosing times throughout the day and restrictions on what medications can take together.^7^ One of the main reasons for the rejection and graft loss is the stated medication nonadherence.^44^, ^45^ Rates of medication nonadherence are reported as high as 65%.^27^ Due to the importance of this issue, all medication adherence studies were considered.

Transplant survival decreases with time, and long‐term survival rates vary significantly depending on the type of transplant and age group. Biological bases in the reduction of patient and graft survival play strong roles, but other factors such as adherence to medical care and the medication regimen are also effective. These factors are less common among pediatric transplant candidates and adolescents. Nearly 45% of all pediatric transplant candidates and recipients are adolescents. The usage of m‐Health to promote the performance of self‐care behaviors among adolescent transplant recipients offers a new approach to solving this problem.^7^ However, few studies have focused only on adolescents. In this research, only two studies of children and adolescents were considered and parents were involved as an active component in the field of m‐Health.^7^, ^39^

In addition, children and adolescents have many psychosocial consequences in the COVID‐19 epidemic, with lengthy school closures, disruption of daily schedules, and peer interactions around the world. People with chronic health conditions, such as solid organ transplant recipients who have already experienced a psychosocial burden, are more likely to experience more consequences such as increased anxiety about health, academic and social challenges associated with school closures, and increased risk of family stress. Now more than ever, digital approaches to optimize the delivery of health care for children and adolescents have become important. Smartphones are one of the favorite technologies for children and teenagers available to almost all of them. Therefore, m‐Health can be used to reduce psychosocial consequences and improve self‐management skills.^46^, ^47^, ^48^

Telemedicine is defined as “remote delivery of healthcare services over the telecommunication infrastructure.” Concepts of telemedicine with web‐based platforms, mobile applications, video conferencing, chat, remote vital sign monitoring, or a variety of those combinations are suitable for transplantation follow‐up. However, the additional advantage of new telemedicine concepts over standard posttransplant care must be demonstrated to justify the use of additional costs.^49^ The development and implementation of these systems entail several different costs such as the cost of equipment, personnel, and communications.^50^ Two of the most common methods of economic evaluation are cost‐utility analysis (CUA) and cost‐effectiveness analysis (CEA). CUA is especially used in the evaluation of health technology. The main purpose of the CUA is to estimate the cost ratio of a health‐related intervention and its benefits in terms of the number of years that users live in perfect health.^50^, ^51^ Today, the introduction of new e‐Health technologies significantly increases the cost‐effectiveness of the healthcare system.^52^ Although m‐Health is a cost‐effective one of telemedicine technology with a low‐cost and efficient strategy, the evidence is limited, and most studies have not reported it and lacked a comprehensive analysis.^53^, ^54^ In addition, a study found the development and successful implementation of a m‐Health application costly and time‐consuming.^32^ According to previous studies, the main limitations of the economic evaluation of telemedicine are the lack of randomized controlled trials, the size of small samples, and the lack of quality data and appropriate measures.^50^

To develop effective m‐Health applications to support self‐management, end‐user participation is essential, and previous research has shown that end‐user engagement leads to higher levels of user acceptance and satisfaction. User satisfaction when using m‐Health applications as a tool to support self‐management is of particular importance. Because satisfaction reduces the barriers to successful implementation.^7^

Lack of usability can be a major barrier to the rapid adoption of mobile services.^55^ Most of the studies reviewed in this study did not use standard usability tools to evaluate the program, and most applications reach consumers with little to no empirical evaluation, but Pocket PATH is one of a limited number of m‐Health interventions to have undergone user‐centered development and testing.^35^

The unavailability of the studied mobile health applications in the application market is considered a limitation in our study because their installation could be useful in order to more closely examine the capabilities of applications and evaluation.

## 5 CONCLUSION

We conducted a comprehensive review of m‐Health applications in posttransplant care. We found that m‐Health solutions can help the patient in self‐management in many ways after transplantation. Medication management is important after solid organ transplantation, and according to the evidence, this study shows that m‐Health in this field strengthens medication adherence and may help empower patients. For more exploitation and better results, it is suggested that more attention be paid to understanding the end‐user's expectations and participation in developing an m‐Health application; also more attention should be paid to user design and usability factors.

## CONFLICT OF INTEREST

The authors declare no conflicts of interest.

## AUTHOR CONTRIBUTIONS

Conceptualization: Sanaz Abasi, Azita Yazdani.

Data curation: Sanaz Abasi, Shamim Kiani

First Draft Preparation: Sanaz Abasi, Shamim Kiani

Review and Editing: Azita Yazdani, Zahra Mahmoudzadeh‐Sagheb

Supervision: Azita Yazdani, Zahra Mahmoudzadeh‐Sagheb

All authors have read and approved the final version of the manuscript.

Zahra Mahmoudzadeh‐Sagheb had full access to all of the data in this study and take complete responsibility for the integrity of the data and the accuracy of the data analysis.

## TRANSPARENCY STATEMENT

Zahra Mahmoudzadeh‐Sagheb and Azita Yazdani affirm that this manuscript is an honest, accurate, and transparent account of the study being reported; that no important aspects of the study have been omitted; and that any discrepancies from the study as planned (and, if relevant, registered) have been explained.

## Data Availability

All data analyzed for and presented in this article are from the 16 studies we reviewed. The data are accessible via referenced articles.
